# A Medically Managed Case of Acromegaly: A Case Report

**DOI:** 10.7759/cureus.101136

**Published:** 2026-01-09

**Authors:** Karam Bdour, Khaldon Al-Sarihin, Nesreen El Issa, Odai Alwraikat, Mohammad Albadarneh, Rania Al-Asa'd, Mu'taz Alwadi

**Affiliations:** 1 Endocrinology and Diabetes, Jordanian Royal Medical Services, Amman, JOR; 2 Opthalomology, Jordanian Royal Medical Services, Amman, JOR

**Keywords:** acromegaly, dopamine agonists, gigantism, growth hormone, growth hormone-secreting pituitary adenoma, insulin-like growth factor 1, pituitary adenoma, somatostatins analogues, somatotroph adenoma

## Abstract

Endocrine glands are specialized cells responsible for producing and secreting hormones that serve many functions in various body tissues. The pituitary gland is an endocrine gland located in the brain, composed of anterior and posterior parts, both of which are responsible for the secretion of many hormones. The anterior pituitary gland secretes six hormones. Hypersecretion and undersecretion result in abnormalities in the body. Autonomous growth hormone production due to a pituitary adenoma leads to various changes in tissues and organs. Excess growth hormone results in gigantism during childhood, while in adulthood, it is called acromegaly. In this report, we present a 39-year-old man with a large somatotroph macroadenoma, which was managed entirely with medical therapy, resulting in significant biochemical and anatomical improvement. This case highlights the potential role of combined somatostatin analogs and dopamine agonists in selected patients with acromegaly.

## Introduction

The anterior part of the pituitary gland is responsible for the formation and release of six hormones, each of which has a specific function. The blood levels of these hormones are orchestrated based on positive and negative feedback [1,2]. The uncontrollable release of growth hormone (GH) results in gigantism or acromegaly. Acromegaly is a disease characterized by many clinical manifestations that occur in adulthood (after bony epiphyseal plates fusion) due to excessive GH secretion [1,3,4].

Acromegaly has a prevalence of between 2.8 and 13.7 cases per 100,000 population, and it has no sex difference; the average age of diagnosis is around forty [5,6].

The most common cause of this disease is a pituitary adenoma with excess secretion of GH. Rare cases of GH excess can be observed in neuroendocrine tumors, which produce GH-releasing hormone (GHRH), stimulating uncontrollable GH release from the pituitary gland [7].

Diagnosing acromegaly is suspected when there is a high clinical suspicion based on many features, such as an increase in the size of body tissues.

Acromegaly should be diagnosed biochemically and structurally by a high insulin-like growth factor 1 (IGF1) level, along with clinical features, and further workup, including an oral glucose tolerance test (OGTT) for GH and the presence of a pituitary adenoma on pituitary imaging using contrast-enhanced magnetic resonance imaging [7].

As GH and prolactin both originate from somatotroph cells, some cases of GH excess have prolactin co-secretion. Around a quarter of patients diagnosed with acromegaly have prolactin hormone excess [8-10].

Once confirmed biochemically and structurally, the gold standard treatment is transsphenoidal adenectomy [3,7].

## Case presentation

We represent Mr. X, a 39-year-old male who presented to the endocrine clinic at King Hussain Medical Center after an incidental diagnosis of pituitary adenoma during evaluation of chronic headaches. Consent was taken from the patient to publish his case, but he did not accept publishing personal images.

Upon evaluation, his blood pressure was 141/83 with a normal temperature and heart rate. He had coarse facial features, protrusion of the mandible, macroglossia, a mild, smooth, non-nodular goiter, and bulky hands. His visual fields were assessed using a confrontation test, which revealed the presence of bitemporal hemianopia. After taking a detailed history, he noted that he had changed his shoe size from 42 to 44 over the last three years; additionally, he reported experiencing a long-standing headache accompanied by diminished vision. When asked about his sexual life, he reported experiencing low libido and erectile dysfunction. The patient reported that his wedding ring no longer fit due to increased finger size.

His vision was assessed by visual perimetry, and bitemporal hemianopia was also shown.

A provisional diagnosis of acromegaly was suspected. Thus, a proper workup was initiated, including laboratory and radiological tests.

Laboratory and radiological diagnosis

The hormonal panel of the pituitary gland indicated elevated levels of GH and IGF-1. The IGF-1 level was 742 ng/mL, and the GH level was 22.7 ng/mL. Prolactin was 440 ng/mL, thyroid-stimulating hormone (TSH) was 2.1 µIU/mL, cortisol was 8.9 (µg/dL), and adrenocorticotropic hormone (ACTH) was 39 (pg/mL). Both luteinizing hormone (LH) and follicle-stimulating hormone (FSH) were at low normal levels (mIU/mL).

Free T4 (µg/mL) level was 0.6, and total testosterone level was around 180 µg/dL in two morning readings (normal range 246-836 ng/dL). Regarding blood sugar, his HbA1c was 6.4% and fasting blood sugar was 115 mg/dL.

According to the 2014 Endocrine Society Clinical Practice Guideline on acromegaly, the gold standard diagnostic test is the lack of suppression of GH to < 1 ng/mL after ingesting 75 g of the OGTT. Consequently, the patient underwent the OGTT, and it confirmed the diagnosis of acromegaly [3]. Table 1 shows the OGTT result.

**Table 1:** Oral glucose tolerance test results Oral glucose tolerance test for growth hormone.

75 g oral glucose tolerance test (min)	Baseline	30	60	90	120
Growth hormone (ng/mL)	21.5	8.7	6.1	5.2	10.5
Blood glucose (mg/mL)	81	130	111	126	146

A two-dimensional transthoracic echocardiography showed mild left ventricular hypertrophy with normal ejection fraction and no valvopathy.

**Figure 1:**
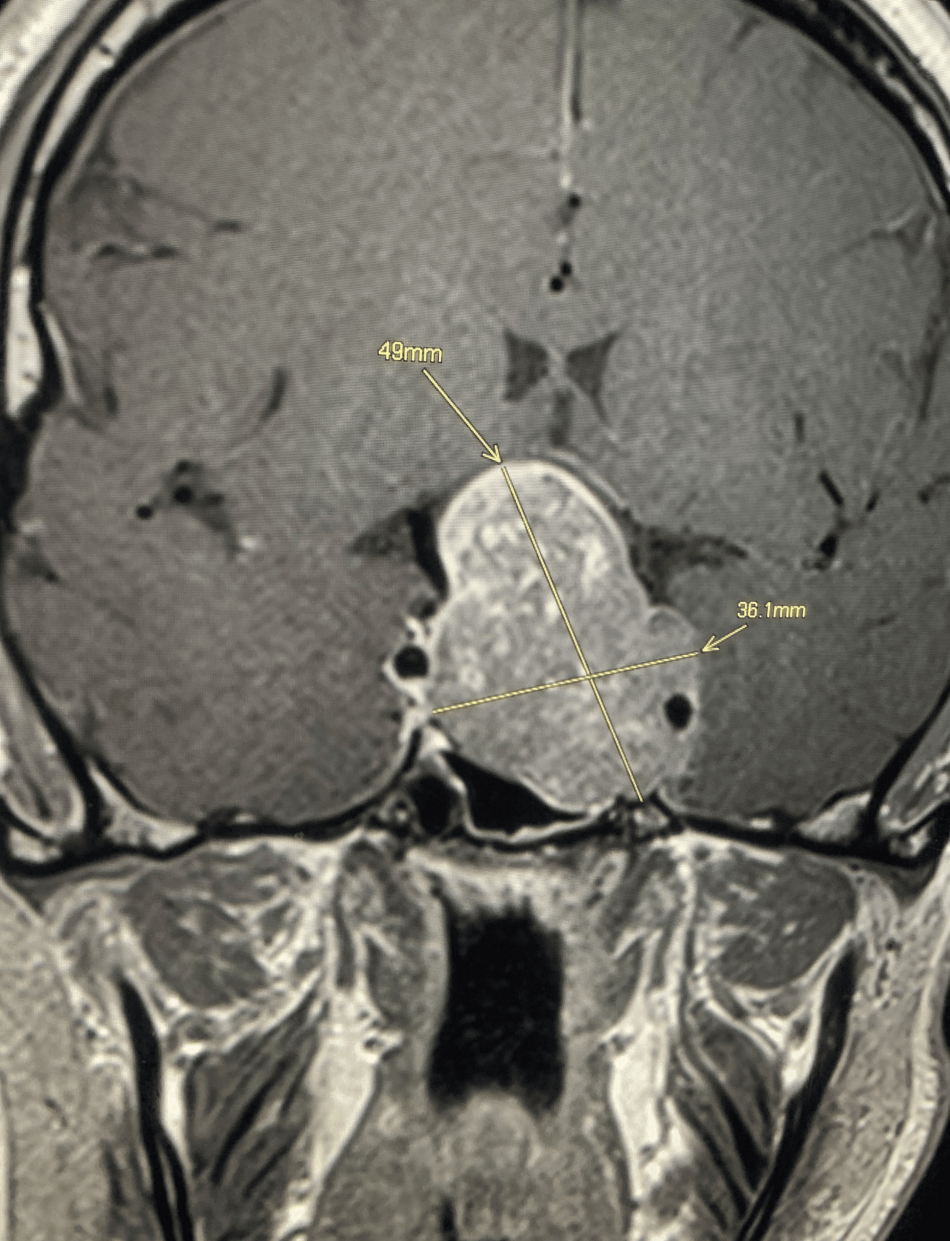
Pituitary MRI - coronal view Before the treatment, showing the initial dimensions of the lesion.

**Figure 2:**
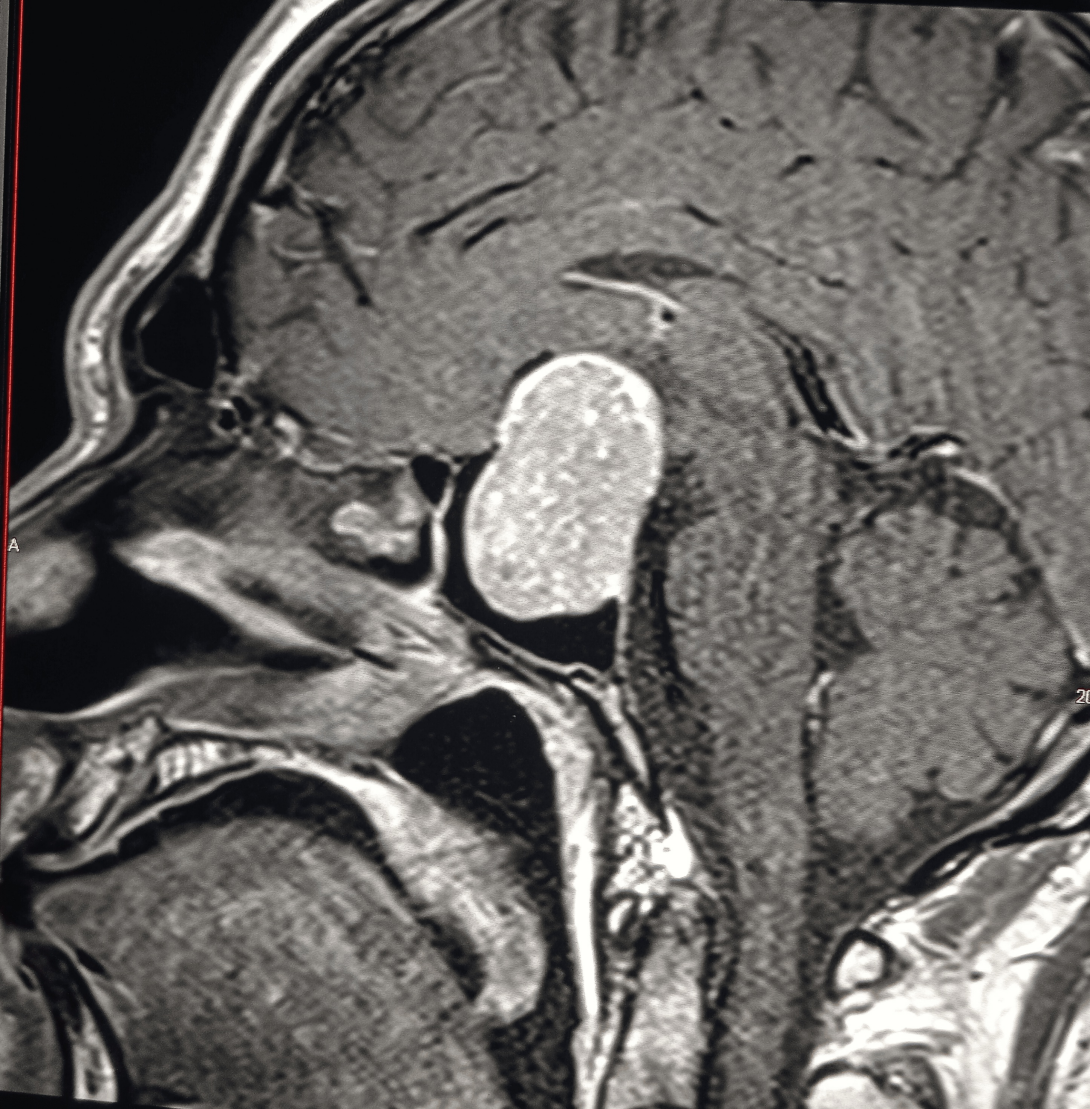
Pituitary MRI - sagittal view Before the treatment.

Magnetic resonance imaging of the pituitary gland revealed a very large macroadenoma measuring 48 × 38 × 29 mm, with hemorrhagic components pushing the optic chiasm superiorly and extending both inferiorly and superiorly, while laterally invading the cavernous sinuses (Figures 1-2).

Treatment

Treatment lines were discussed with the patient. This includes initially surgical treatment as the gold standard treatment, medical treatment (somatostatin analogues, pegvisomant, and dopamine receptor agonists) as the second line, and radiotherapy as the third line [3,5].

As surgical management is the first line of treatment in such cases, the patient was given the first dose of somatostatin analogues, had the low free T4 level replaced with thyroxine 75 mcg/day, and was referred for neurosurgery for the surgical option.

The procedure, course, and outcomes were reviewed with the patient, and an experienced surgeon advised the transcranial approach owing to the large size of the macroadenoma and its extension into adjacent tissues. However, the patient declined surgery due to perceived risks and was hesitant to undergo surgery via the transcranial approach.

The patient was referred back to the endocrine clinic for a different management module. The medical treatment options in the patient's case were discussed, and the medical plan was 120 mg lanreotide long-acting release (LAR) monthly via deep subcutaneous injection [3,5]. Due to the presence of co-secretion, a dopamine agonist was added to his treatment plan [11]. Using cabergoline 2 mg per week. Because of the huge size of his macroadenoma, pegvisomant was avoided as it might cause tumor growth and put more pressure on adjacent tissues [12].

The patient was periodically assessed using imaging and a biochemical profile during his follow-up. His vision had improved more markedly peripherally, and some of his symptoms had disappeared. Such as the resolution of his headache, better sexual drive and activity, and he also observed that his hands were getting smaller. His blood pressure was around 110/70 during multiple clinic visits.

**Figure 3:**
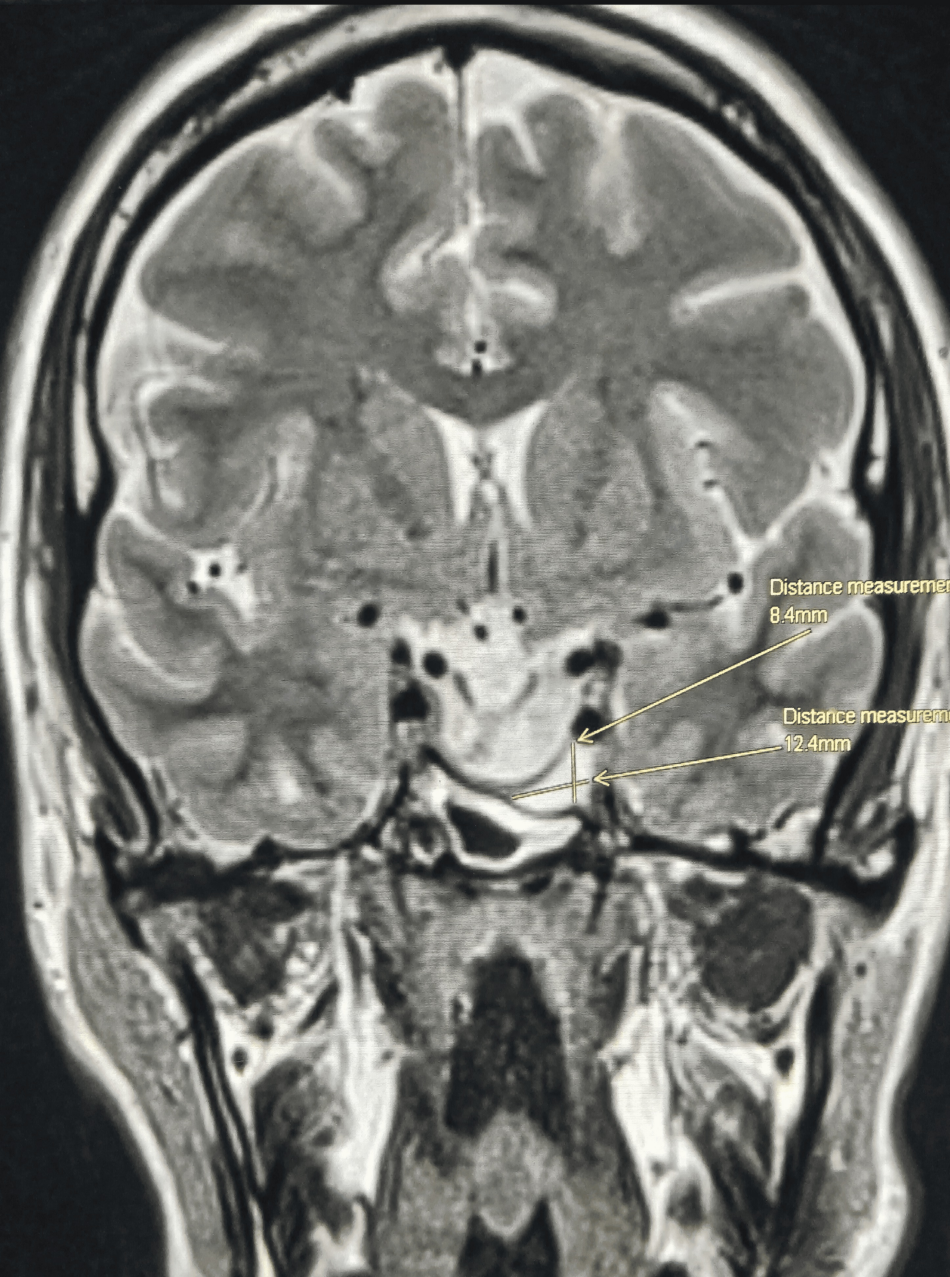
Pituitary MRI - coronal view Twelve months after treatment, with the dimensions of lesion.

**Figure 4:**
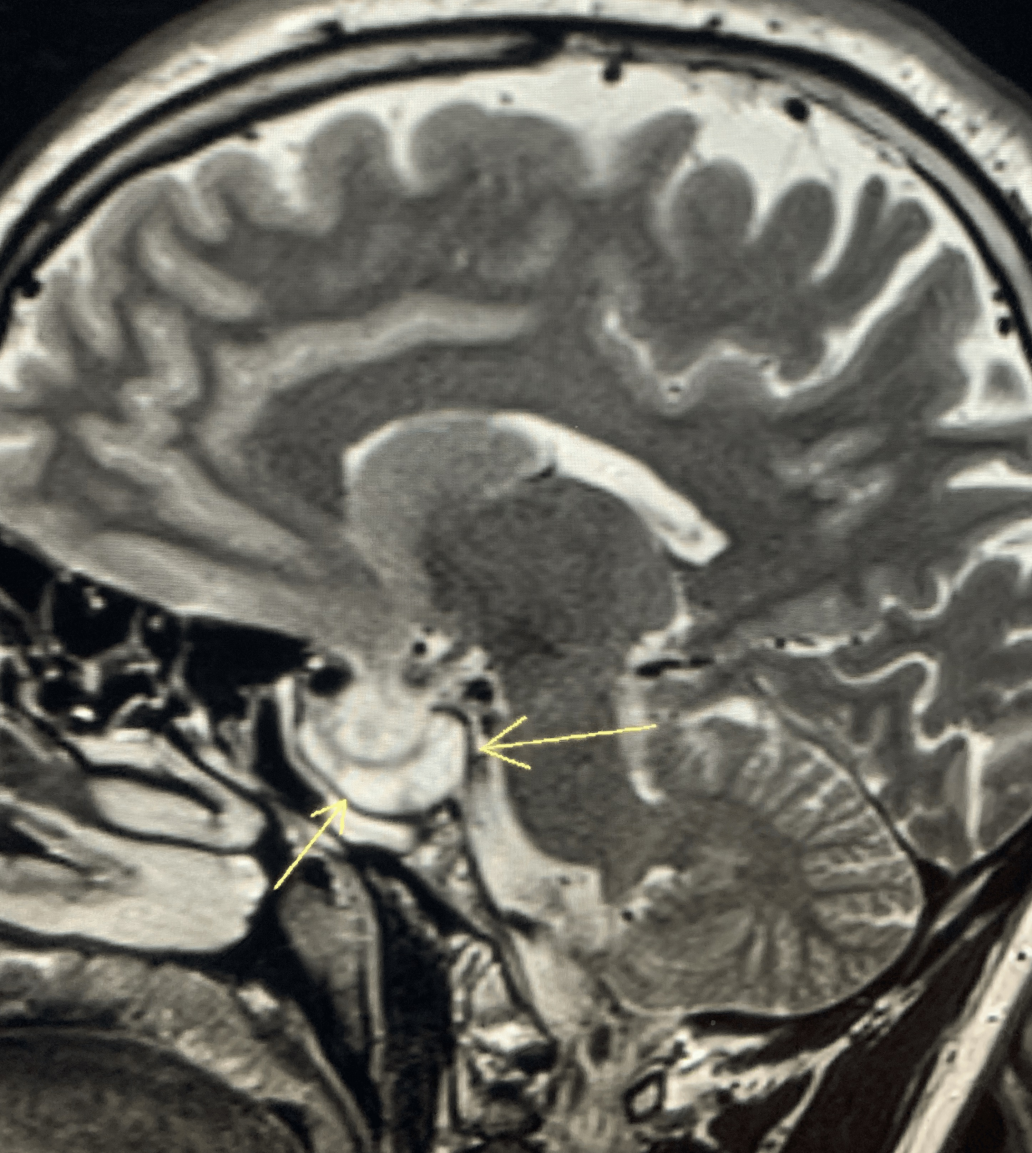
Pituitary MRI - sagittal view Twelve months after treatment.

**Figure 5:**
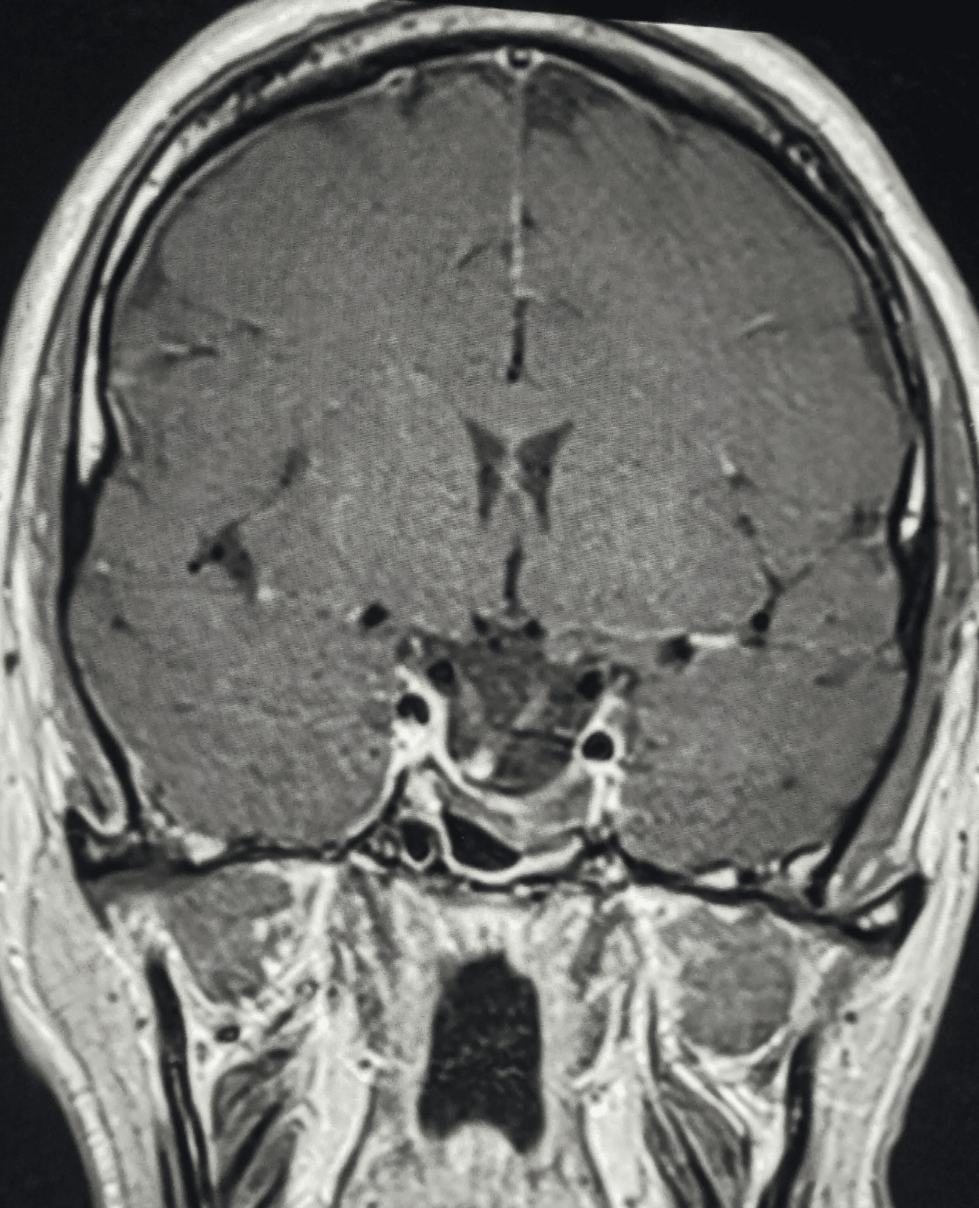
Pituitary MRI - coronal view Twelve months after treatment.

After 12 months of medical treatment, the size of his pituitary macroadenoma decreased by almost 75%, measuring 12 × 10 × 8 mm abutting the optic chiasm. The patient’s IGF1 dropped to 253, and prolactin to 10.8 ng/dL. Figures 3-5 demonstrate the shrinkage in size. 

The dramatic improvement in his symptoms and his IGF1/prolactin levels, along with structural regression of tumor size, was all achieved by medical treatment only [13-15].

There are some cases reported in the literature for successful shrinkage of somatotroph macroadenoma, but they needed more time [16-20]. Probably the addition of a dopamine agonist to the medical management helped to enhance the structural shrinkage of the somatotroph adenoma and shortened the duration needed for medical success in this case [11,21,22]. Table 2 shows the hormonal changes during the course of management.

**Table 2:** Laboratory table before and after the medical treatment GH, growth hormone; IGF-1, insulin-like growth factor 1; TSH, thyroid-stimulating hormone; ACTH, adrenocorticotropic hormone; LH, luteinizing hormone; FSH, follicle-stimulating hormone; FBS, fasting blood sugar

	Initial levels	Three months post-treatment	Six months post-treatment	12 months post-treatment	Normal values
GH	22.7	15.7	13.9	4.5	0.06-5 ng/mL
IGF-1	784	567	343	256	220-307 ng/mL
Prolactin	440	276	157	17.6	4-15 ng/mL
TSH	1.33	1.59	1.4	1.07	0.27-4.2 µIU/mL
T4	0.6	1.3	1.4	1.2	0.7-1.7 ng/dL
ACTH	57	38.8	38	47.6	7.2-63 pg/mL
Cortisol	8.25	12.6	11.67	13.8	7.0-25 ug/dL
LH	2.1	2.7	3.6	3.5	1.3-9.6 mIU/mL
FSH	1.8	1.5	1.9	3.1	1.2-15.8 mIU/mL
Total testosterone	73.6	49	201	278	246-836 ng/dL
HbA1c	6.4%	6.1%	5.8%	5.7%	Normal < 5.6%
FBS	111	105	106	98	90-100 mg/dL

## Discussion

Acromegaly is an uncommon disorder with gradual manifestations; usually, the patient presents due to acromegaly's systemic complications in the body organs [3]. Acromegaly is a slow-developing condition that can go unnoticed for a long time, which often results in a late diagnosis or treatment.

Over the years, the sustained hypersecretion of GH has resulted in cardiovascular compromise, respiratory dysfunction, higher malignancy risk, metabolic disorders, skeletal deformities, arthropathy, and neuropathies. Therefore, it is not unusual for the consequences of acromegaly to increase mortality in affected individuals [7]. The cardiovascular changes (cardiomyopathy) account for the major causes of mortality [23]. That is why most acromegaly patients present to different medical specialties before the disease is diagnosed [24].

Acromegaly is mostly caused by pituitary adenoma [2-4,7]. Some rare causes result from ectopic secretion of GH or GHRH by different tumors [7,25]. Prolactin is co-secreted in approximately 25% of individuals with acromegaly [8-10].

When diagnosed, a GH-secreting pituitary adenoma is the main cause of acromegaly. Based on tumor size and the presence of optic chiasm compression, visual field disturbances may occur along with systemic manifestations of GH oversecretion [3,7].

Adenectomy, specifically via the transsphenoidal approach, appears to be the initial treatment for most patients. Suppose surgery is not considered as a primary treatment, such as in patients with advanced age, fragile medical status, or the patient’s preference [26]. Medical therapy, including somatostatin analogs, can result in adequate treatment by suppressing the autonomous secretion of GH with subsequent inhibition of IGF-1 production by the liver and controlling the effects of GH hypersecretion on various body tissues [13-15]. Pegvisomant is also used as part of the medical management of acromegaly; it blocks the receptor of GH, which, as a result, decreases the IGF-1 level. However, such a mechanism can increase the size of the pituitary adenoma and might put more compression on the surroundings of the pituitary gland [12]. Dopamine agonists can also be part of the medical treatment of acromegaly [11,21,22]. The use of dopamine agonists showed marked control in cases of acromegaly manifested by normalization of IGF-1 level and mass reduction of the pituitary adenoma, especially in prolactin co-secreting adenomas [22-29].

The term silent pituitary adenoma is not applicable in this case because the patient had overt acromegaly manifested clinically and biochemically, as the GH and IGF-1 were both elevated throughout the disease management [30].

Mr. X presented with overt acromegalic features, and after proper and targeted assessment, the acromegaly was confirmed biochemically. The MRI of the pituitary gland revealed a large pituitary adenoma compressing the adjacent structures.

In this case report, we highlight the outcome of medical treatment in an acromegaly patient, and how it resulted in structural effects on pituitary adenoma size, and biochemical normalization of IGF-1 level and prolactin level.

As the patient was reluctant to undergo adenectomy, the medical treatment was the next option according to the treatment guidelines [3,5]. The use of somatostatin analogues along with a dopamine agonist for 12 continuous months showed a significant decrease in somatotroph adenoma size, and the IGF1 concentration reached a normal level. The prolactin level also dropped to 15 ng/dL.

The patient's clinical features of acromegaly syndrome have almost resolved completely; however, he still has some facial coarsening. The patient had successful management using medical modalities only.

Limitations include the single-patient nature of the report and relatively short follow-up, but the findings add to growing evidence supporting medical therapy as a viable primary option in selected cases.

## Conclusions

Acromegaly is frequently diagnosed late due to its slow progression and nonspecific manifestations. When a pituitary adenoma is the cause, transsphenoidal surgical resection remains the cornerstone of treatment, while medical therapy is generally reserved for specific circumstances, such as patient refusal or persistent disease after surgery.

This case demonstrates that in selected patients, an exclusive medical therapy with lanreotide and cabergoline can achieve excellent biochemical control and meaningful tumor shrinkage. Timely diagnosis, individualized treatment planning, and close follow-up are essential to optimize outcomes and reduce long-term complications of GH excess. Larger studies and longer follow-up are needed to confirm these findings, but this case adds to the growing evidence that individualized medical management can be both safe and effective in acromegaly.

## References

[1] Sadiq NM, Tadi P (2023). Physiology, pituitary hormones. StatPearls.

[2] Perez-Castro C, Renner U, Haedo MR, Stalla GK, Arzt E (2012). Cellular and molecular specificity of pituitary gland physiology. Physiol Rev.

[3] Katznelson L, Laws ER Jr, Melmed S, Molitch ME, Murad MH, Utz A, Wass JA (2014). Acromegaly: an endocrine society clinical practice guideline. J Clin Endocrinol Metab.

[4] Bello MO, Garla VV (2025). Gigantism and acromegaly. StatPearls.

[5] Ogedegbe OJ, Cheema AY, Khan MA (2022). A comprehensive review of four clinical practice guidelines of acromegaly. Cureus.

[6] Lavrentaki A, Paluzzi A, Wass JA, Karavitaki N (2017). Epidemiology of acromegaly: review of population studies. Pituitary.

[7] Ben-Shlomo A, Melmed S (2008). Acromegaly. Endocrinol Metab Clin North Am.

[8] Van Laethem D, Michotte A, Cools W, Velkeniers B, Unuane D, Andreescu CE, Bravenboer B (2020). Hyperprolactinemia in acromegaly is related to prolactin secretion by somatolactotroph tumours. Horm Metab Res.

[9] Huan C, Cui G, Ren Z (2015). The characteristics of acromegalic patients with hyperprolactinemia and the differences with hyperprolactinemia patients. Pak J Pharm Sci.

[10] Wang M, Mou C, Jiang M (2012). The characteristics of acromegalic patients with hyperprolactinemia and the differences in patients with merely GH-secreting adenomas: clinical analysis of 279 cases. Eur J Endocrinol.

[11] Biagetti B, Araujo-Castro M, Torre EM (2024). Effectiveness of combined first-line medical treatment in acromegaly with prolactin cosecretion. Eur J Endocrinol.

[12] Buhk JH, Jung S, Psychogios MN (2010). Tumor volume of growth hormone-secreting pituitary adenomas during treatment with pegvisomant: a prospective multicenter study. J Clin Endocrinol Metab.

[13] Störmann S (2022). Let's focus more on regional diversity of acromegaly. Ann Transl Med.

[14] Freda PU, Katznelson L, van der Lely AJ, Reyes CM, Zhao S, Rabinowitz D (2005). Long-acting somatostatin analog therapy of acromegaly: a meta-analysis. J Clin Endocrinol Metab.

[15] Colao A, Grasso LF, Giustina A, Melmed S, Chanson P, Pereira AM, Pivonello R (2019). Author correction: acromegaly. Nat Rev Dis Primers.

[16] Colao A, Ferone D, Marzullo P (2001). Long-term effects of depot long-acting somatostatin analog octreotide on hormone levels and tumor mass in acromegaly. J Clin Endocrinol Metab.

[17] Kurahashi K, Endo I, Kondo T (2017). Remarkable shrinkage of a growth hormone (GH)-secreting macroadenoma induced by somatostatin analogue administration: a case report and literature review. Intern Med.

[18] Resmini E, Murialdo G, Giusti M, Boschetti M, Minuto F, Ferone D (2005). Pituitary tumor disappearance in a patient with newly diagnosed acromegaly primarily treated with octreotide LAR. J Endocrinol Invest.

[19] Harinarayan CV (2004). Primary octreotide LAR therapy in GH-secreting pituitary adenoma. J Indian Med Assoc.

[20] Ozbek M, Erdogan M, Akbal E, Gönülalan G (2009). Disappearance of a GH secreting macroadenoma, during long-term somatostatin analogue administration. Exp Clin Endocrinol Diabetes.

[21] Gatto F, Feelders RA, van Koetsveld PM (2023). Dissecting the in vitro efficacy of octreotide and cabergoline in GH- and GH/PRL-secreting pituitary tumors. J Clin Endocrinol Metab.

[22] Kuhn E, Chanson P (2017). Cabergoline in acromegaly. Pituitary.

[23] Orme SM, McNally RJ, Cartwright RA, Belchetz PE (1998). Mortality and cancer incidence in acromegaly: a retrospective cohort study. United Kingdom Acromegaly Study Group. J Clin Endocrinol Metab.

[24] Wang K, Guo X, Yu S (2021). Patient-identified problems and influences associated with diagnostic delay of acromegaly: a nationwide cross-sectional study. Front Endocrinol (Lausanne).

[25] Adigun OO, Nguyen M, Fox TJ (2023). Acromegaly. StatPearls.

[26] Melmed S, Colao A, Barkan A (2009). Guidelines for acromegaly management: an update. J Clin Endocrinol Metab.

[27] Abs R, Verhelst J, Maiter D (1998). Cabergoline in the treatment of acromegaly: a study in 64 patients. J Clin Endocrinol Metab.

[28] Yalçın MM, Keser GB, Coşkun M (2024). Long-term results of cabergoline add-on long-acting somatostatin analogue therapy in acromegaly patients. Gazi Med J.

[29] Sandret L, Maison P, Chanson P (2011). Place of cabergoline in acromegaly: a meta-analysis. J Clin Endocrinol Metab.

[30] Wade AN, Baccon J, Grady MS, Judy KD, O'Rourke DM, Snyder PJ (2011). Clinically silent somatotroph adenomas are common. Eur J Endocrinol.

